# Chicago College of Dental Surgeons

**Published:** 1888-07

**Authors:** 


### Chicago College of Dental Surgery.

The sixth annual commencement of the Chicago College of Dental Surgery was held at the Grand Opera House, Chicago, Ill., on Tuesday, March 27, 1888, at 2.30 P. M.

The degree of D.D.S. was conferred on the following graduates by Dr. James A. Swasey, President of the College:

John Wesley Alderson.
John Charles Barclay.
George Heinrich Becker.
Clayton William Bennett.
Orrin George Bennett.
Frank William Cady.
Sherman Lee Chappell.
Frank Beaumont Clarke.
Rush Eugene Crissman.
William Gould Dalrymple.
Charles Henry Darling.
Frank Henry Davis.
Samuel Finley Duncan.
William Andrew Fortuin.
Clarence Barnard Freeman.
Robert Curtis Gardner.
Thomas Dimma Granner.
Grant Arthur Goodrich.
Valentine Arthur Gudex.
Alfred Ward Hebert.
Peter Monroe Hendershott.
Albert Frank Henkel.
Thomas Francis Henry.
Richard Herrmann.
James Ward House.
Henry K. Kerman.
Richard Kessel
William Kuester.
Louis Frank Lattan.
George Edward Long.
Alfred Lowther.
Anthony Mann.
Clare Winchel Marshall.
Edward Martin McIntosh.
Charles James Merriman
Ewing Van Darian Morris, M.D.
Hans Theodore Nordahl.
Adelbert Henry Peck.
George Reedy.
Frank M. Russell.
Harry Reid Staley.
Henry Stewart.
Rupert DeGeorge Treen.
Samuel Adolphus Whedon.

